# Ethanol Effects on Early Developmental Stages Studied Using the Zebrafish

**DOI:** 10.3390/biomedicines10102555

**Published:** 2022-10-13

**Authors:** Priyadharshini Manikandan, Swapnalee Sarmah, James A. Marrs

**Affiliations:** Department of Biology, School of Science, Indiana University-Purdue University Indianapolis, Indianapolis, IN 46202, USA

**Keywords:** fetal alcohol spectrum disorder, ethanol, zebrafish, development, gastrulation, *sox2*, *elf3*, *shh*

## Abstract

Fetal alcohol spectrum disorder (FASD) results from prenatal ethanol exposure. The zebrafish (*Danio rerio*) is an outstanding in vivo FASD model. Early development produced the three germ layers and embryonic axes patterning. A critical pluripotency transcriptional gene circuit of *sox2*, *pou5f1* (*oct4*; recently renamed *pou5f3*), and *nanog* maintain potency and self-renewal. Ethanol affects *sox2* expression, which functions with *pou5f1* to control target gene transcription. Various genes, like *elf3*, may interact and regulate *sox2*, and *elf3* knockdown affects early development. Downstream of the pluripotency transcriptional circuit, developmental signaling activities regulate morphogenetic cell movements and lineage specification. These activities are also affected by ethanol exposure. Hedgehog signaling is a critical developmental signaling pathway that controls numerous developmental events, including neural axis specification. Sonic hedgehog activities are affected by embryonic ethanol exposure. Activation of sonic hedgehog expression is controlled by TGF-ß family members, Nodal and Bmp, during dorsoventral (DV) embryonic axis establishment. Ethanol may perturb TGF-ß family receptors and signaling activities, including the sonic hedgehog pathway. Significantly, experiments show that activation of sonic hedgehog signaling rescues some embryonic ethanol exposure effects. More research is needed to understand how ethanol affects early developmental signaling and morphogenesis.

## 1. Fetal Alcohol Spectrum Disorder

Alcohol is a common teratogen that causes adverse effects during pregnancy. Fetal alcohol spectrum disorder (FASD) covers a range of developmental defects and disorders of prenatal alcohol exposure (PAE), which occur when a woman consumes alcohol during their pregnancy [1]. The consequences of PAE are dependent on many factors, including, amount and duration of alcohol exposure, maternal and fetal age and genetics [1]. Premature death of the fetus also occurs with PAE [1]. Fetal alcohol syndrome (FAS) is the most severe form of the spectrum for babies born following PAE [1]. FAS is characterized by a set of craniofacial dysmorphology, neural defects, cardiac defects, sensory dysfunction, motor disabilities, and learning disabilities [2]. A recent study reported that globally an estimated 1700 babies are born every day with FASD [3]. There is a higher prevalence of FASD cases in higher-risk populations, such as those with a lower socioeconomic status [3]. A study conducted by the Center for Disease Control and Prevention (CDC), using data collected from pregnant women between 2015 and 2017, showed that one in nine women drank at least one alcoholic drink in the past month while pregnant, and around one third of these women reported binge drinking (drinking at least four alcoholic drinks in one sitting) [4]. Due to social biases against pregnant women consuming alcohol, there may be an underreporting of prenatal alcohol exposure incidences [4].

There is no cure for FASD, although treatments have been developed to help symptoms and aid in the development of a child with FASD [5]. Although folic acid has been shown to lessen the effects of early ethanol exposure in mouse, chicken and zebrafish embryos [5,6,7], it is not known whether folic acid protects the human baby from the deleterious effects of alcohol exposure. The only way to avoid FASD is through prevention, by abstaining from alcohol during pregnancy. Educating people about the consequences of FASD on a person’s quality of life may help with prevention [5].

## 2. Use of Zebrafish as an FASD Model

The zebrafish (*Danio rerio*) is an established model for developmental studies of ethanol exposure effects, recapitulating FASD phenotypes [8]. Mammalian models, such as mice, are more similar to human development, but in utero development is difficult to study, particularly early developmental stages [8]. Zebrafish can produce hundreds of fertilized eggs per mating, allowing many embryos to be studied. Zebrafish development is very rapid. Early development, somitogenesis and establishment of the body plan occurs in 24 h. External fertilization eliminates ongoing parental influence during development and allows direct observation of embryos. Embryos and larvae are transparent, facilitating observation. Zebrafish also share extensive genetic evolutionary conservation with humans. The zebrafish genome has been completely sequenced enabling scientists to create mutations using reverse genetics and study the outcomes. Thus, the zebrafish can model human development and be used to study effects of teratogenic factors, like ethanol [5,9,10].

## 3. Early Zebrafish Development

### 3.1. Blastula Stage

Fertilized zebrafish embryos go through a series of rapid cleavages in the first 3 hours post fertilization (hpf) [11]. Initially, a blastodisc of 16 cells forms a syncytium with the yolk cells, and subsequent cleavages produce cells that are no longer connected, as well as cells that are cytoplasmically connected to the yolk cell (yolk syncytial layer; YSL). Early cleavage stages are directed by maternal transcripts deposited in the oocyte [12,13]. The zygotic genome is activated and midblastula transition occurs at the 1000-cell stage at 3 hpf. Afterwards, cell divisions are slower and asynchronous [11,12,13,14].

Zygotic gene expression activation regulates pluripotency and morphogenesis. The pluripotency gene circuit, its role in zygotic genome activation and pluripotency maintenance will be discussed below. The YSL is formed, which interacts with the overlying embryo and is a critical extraembryonic signaling center [15]. The YSL microtubule and actin cytoskeleton drives blastoderm spreading over the yolk cell. This spreading occurs by thinning and expansion of this cell layer in a process called epiboly [16]. Three processes combine to produce epiboly movements: (i) The blastoderm cells migrate toward the vegetal pole; (ii) Microtubules within the yolk cell pull on the membrane-actin junction with the enveloping layer at the germ ring, dragging this junction toward the vegetal pole; and (iii) Radial intercalation thins the blastoderm cell layers, which expands the cell sheet, spreading it over the yolk cell [16].

Epiboly starts around 4 hpf and is the first morphogenetic event in zebrafish development. As the epiboly process begins, the blastula is patterned by extraembryonic signals from the yolk cell [16]. This patterning establishes a pre-gastrulation fate map and is also a prelude to extensive morphogenesis that occurs at the onset of gastrulation [16,17].

The yolk cell is an extraembryonic tissue that lies beneath the blastoderm. The deep cells are a mass of cells that make the embryo proper. The enveloping layer is one cell thick sheet, enclosing the deep cells [13]. The YSL is a multinucleated syncytium within the yolk cell that forms during the blastula stage and matures by the 10th cell cycle [17]. It does not contribute cells or nuclei to the developing embryo, but the YSL secretes signaling factors that induce germ layer specification, embryo patterning, epiboly, and plays an important role in directing cell movements during gastrulation [13,16,17,18]. The yolk cell also provides critical nutrients during development. An array of genes control signaling from the YSL during embryogenesis, including Nodal and its related proteins, which are required for mesoderm induction and dorsal patterning of the blastoderm [13,18].

### 3.2. Gastrulation

When epiboly reaches the yolk cell equator (50% epiboly), mesoderm and endoderm precursors involute at the cell margins. The endoderm forms a ventral layer, and the mesoderm will populate the space between the endoderm and the ectoderm, which remains at the embryo surface. The embryo continues to elongate by epiboly progression to the vegetal pole, while the germ layer precursors converge on the midline and extend along the anterior-posterior axis, in a process called convergence and extension. These massive cell rearrangements, establishing the body axes, require a series of carefully orchestrated cell movements. Zygotic transcription drives morphogenesis, but the critical genes and activities are only partially understood. Axis specification is coupled with convergence and extension, and thus, the transcriptional mechanisms that specify the anterior-posterior and dorsal-ventral axes work in coordination with the morphogenetic movements that organize the body plan. These gastrulation events are preludes to full establishment of the body plan and organogenesis [16].

### 3.3. Pluripotency Circuit

Previously, zygotic genome activation was thought to be abrupt at midblastula transition. New evidence indicates that there is a progressive series of zygotic genome activation events. In addition to regulating morphogenesis, zygotic genome activation induces and maintains pluripotency [11,14]. One important result of zygotic genome activation is the expression of the pluripotency transcriptional gene circuit: sex-determining region Y-box containing gene 2 (*sox2*), octamer-binding protein 4 (*oct4*) also known as POU domain class 5 transcription factor 1 (*pou5f1,* recently renamed *pou5f3*) in zebrafish, and *nanog* q50 homeobox [14,19,20,21,22]. These transcription factors activate their own and each other’s gene transcription, which produces a self-maintaining, feed-forward circuit that maintains pluripotent stem cell self-renewal and represses differentiation [17,23,24].

Pou5f1 and Sox2 proteins dimerize and work together to activate *nanog* and other pluripotency genes [17]. These genes also participate in the zygotic genome activation, while activating the pluripotency transcriptional program [14,25]. Sox2 has a high mobility group (HMG) DNA-binding domain and a transactivation domain [19]. Sox genes are grouped based on their homology within HMG domains. In the zebrafish, *sox2*, along with *sox1*, *sox3*, and *sox19a/b* are part of the SoxB1 group expressed in the early embryo that share a similarity in sequence and are functionally redundant to one another [24]. During early embryogenesis, maternally provided *sox19b* activates transcription, and *sox2* is one of the first zygotic genes to be transcribed [24,26]. Pou5f1 has two DNA binding domains, a low-affinity POU-specific domain and a higher affinitiy POU-homeodomain [19]. Nanog functions through its one homeodomain that binds to DNA [19]. These factors cooperate to accurately control a critical transcriptional program prior to gastrulation. At gastrulation, this transcriptional circuit is interupted, allowing specification of the 3 germ layers and the initation of appropriate differentiation programs [11,17,19].

## 4. Consequences of Ethanol Exposure during Early Zebrafish Development

Embryos treated with ethanol starting from 2 hpf display defects at early stages, including reduced epiboly progression, which is due to defects in cell adhesion, microtubule organization, and radial intercallation cell movements [6,16,25]. Effects of early ethanol exposure lead to signaling defects that persist and influence later embryogenesis stages [25], but mechanisms remain unclear. Epiboly cell movements are coupled to morphogenesis during gastrulation, and cell adhesion regulation orchestrates these morphogenetic events, particularly convergence and extension of the body axis [6,16]. Cadherin adhesion responds to developmental signaling during gastrulation [16,27]. When E-cadherin was measured, there was little or no change in its expression levels. However, cells in ethanol exposed embryos showed adhesion reduction and morphogenesis changes characteristic of reduced adhesion (radial intercallation and cell migration defects). Convergence and extension gastrulation cell movements also depend on cell intercallation and cell migration events [6].

Morphogenesis defects arise from ethanol exposure. Morphogenesis defects may be caused by ethanol effects on early zygotic gene expression, which regulates some of the earliest morphogenetic events. It is also possible that ethanol has direct biochemical effects on the cytoskeleton, developmental signaling machinery or other components. Ethanol exposure at 2 hpf leads to gastrulation defects due to cell adhesion and microtubule defects, which begin during the blastula stage [6,25]. Ethanol exposure also dysregulates genes that are evolutionarily conserved in the vertebrates and regulated during gastrulation like the reduction in *sox2* expression [6,25].

### 4.1. Pluripotency Gene Expression Defects

#### 4.1.1. *sox2*

Gene expression analysis showed that expression of numerous genes are affected by ethanol exposure. A study on ethanol effects on mouse embryonic stem cell proteins reported that ethanol effects the stoichiometry of SOX2 and OCT4, and it skews the normal functional balance of the two [20]. Pluripotency regulator *sox2* was reduced in the pre-gastrulation zebrafish embryo, which then reduced *sox2* target gene expression [25]. Epiboly and gastrulation cell movements are reduced by ethanol. The pluripotency gene *pou5f1* works with *sox2* and also regulates epiboly and gastrulation cell movements. Injecting small amounts of *sox2* mRNA restores gene expression, epiboly and gastrulation cell movements. A gene-regulatory network affected by ethanol exposure was found that includes sox2 [25]. It is likely that ethanol produces defects through pleiotropic effects on this network, and restoration of normal developmental gene expression would require manipulation of several genes. It could be possible to identify a hierarchy of transcription regulators, making the manipulation easier by controling a small subset of genes that are at the top of the gene regulatory network hierarchy.

#### 4.1.2. *elf3*

The Elf3 (E74 like ETS transcription factor 3) transcription factor is dysregulated by ethanol exposure in the early (4.5 hpf) zebrafish embryo [25]. Little is known about this factor’s function in the early embryo. Elf3 is a member of the E26 transformation-specific family of transcription factors, which play a major role in the development and progression of various types of cancers. Elf3 is also involved during development. In humans, *ELF3* expression was detected in the mid-Carnegie stages [28]. In mice, the expression of *Elf3* was detected after fertilization, which remained high until the blastocyst stage [29]. *Elf3* knockout led to lethality of mice in utero [30], and the pups that survived had defects in small intestine epithelial tissue [30]. Studies showed that ELF3 plays role in terminal differentiation of skin epidermis, epithelia of the cornea, keratinocyte, and T cell differentiation. Our current work on understanding the role of Elf3 during zebrafish development indicates that it is critical for the development of epithelial, mesenchymal, and nervous tissues [31]. The *elf3* gene was among the most strongly dysregulated by ethanol in the early embryo, and the Elf3 transcription factor also targets many other genes, which may act as an important factor in a gene regulatory network dysregulated by ethanol exposure [25]. By dysregulating *sox2* and *elf3* and other ethanol sensitive transcription factors, ethanol exposure may disrupt the crucial balance between pluripotency and differentiation.

The interaction of human ELF3 with the pluripotency regulators SOX2, OCT4, and NANOG has been detected. ELF3 knockdown reduced SOX2 and POU5F1/OCT4 expression, whereas overexpression of ELF3 increased SOX2 and POU5F1 expression in high-grade serous ovarian cancer cells [32]. Human embryonic carcinoma NCCIT cell studies showed that ELF3 is a negative transcriptional regulator of OCT4 and NANOG. ELF3 controls the expression of those genes by directly binding to the promoters of OCT4 and NANOG [33]. The interaction of ELF3 with the pluripotency factors varies depending on the cell- and tissue types. A study manipulating *sox2* and *elf3* during development in the embryos with and without ethanol exposure may shed light on the roles of these genes in the FASD pathogenesis.

#### 4.1.3. *pou5f1*

Maternal and zygotic *pou5f1* mutant (MZ*spg*) embryos showed defects in epiboly progression and in all three embryonic lineages [34,35]. Studies have shown, at this stage of development, *pou5f1* determines pluripotency, and then, *pou5f1* facilitates cellular reorganization, cytoskeletal reorganization, migration, and cell adhesion [27,34,35]. The *pou5f1* mutants have defects in the enveloping layer, deep layer cells, and the YSL [27]. Pou5f1 activates Rab5c-mediated endocytosis and recycling, which controls E-cadherin (Cdh1) dynamics during cell migration [36]. Cdh1 loss-of-function produces epiboly defects [37,38], and this phenotype resembles ethanol-treated embryos [6].

### 4.2. Epiboly Defects

The epiboly defect induced by ethanol exposure raised the hypothesis that ethanol reduced Cdh1 expression in the early embryo. However, measuring mRNA and protein levels showed no difference between control and ethanol-treated embryos [6]. We next examined known mechanisms of epiboly: yolk cell microtubule cytoskeleton; radial intercalation cell movements; and cell migration of deep cells [6].

Yolk cell microtubules connect with the leading edge of the embryo (germ band) where the enveloping layer and deep cells adhere to the yolk cell via cadherins. The yolk cell microtubules produce forces that drag this adhesive connection toward the vegetal pole during epiboly. Ethanol exposure from 2–3 hpf fragmented the yolk cell microtubule network, which may affect the forces pulling the embryo toward the vegetal pole as illustrated by the shapes of enveloping layer cells at the adhesive border [6].

Radial intercalation of deep cells occurs when cells at the interior move from the interior layers to the inner and outer surfaces of the deep cell layer, intercalating interior cells between the surface cell layers. These cell movements reduce the deep cell layer thickness and expand the dimensions of this cell sheet, spreading over the yolk cell during epiboly. Ethanol exposure reduced the frequency of radial intercalation events and increased the number of failed intercalation events, where cells move to the surface and then moved back to the interior, reducing epiboly [6].

Directed cell movements toward the vegetal pole and involution during gastrulation are coupled with epiboly and promoting normal convergent extension of the body axis. We tracked cell movements in time-lapse and measured their directionality. Calculating the meandering index showed that cells in ethanol-exposed gastrulating embryos had increased meandering. Furthermore, the shape of the embryonic axis (axial mesendoderm stained using *ntl* probe in situ hybridization) was shorter, wider, and wedge-shaped in ethanol exposed embryos at mid-gastrulation (8 hpf). These data indicate that directed cell movements and convergent-extension cell movements were affected by ethanol exposure [6].

These effects on epiboly (radial intercalation cell movements; and cell migration of deep cells) and gastrulation (convergent extension) phenocopy Cdh1 loss-of-function during early zebrafish development, which prompted us to examine Cdh1 expression levels and distribution. Indeed, we measured a reduced adhesion activity in blastomeres from ethanol exposed embryos in comparison to untreated embryos. Reduced adhesion occurred despite our results showing that there was no reduction in mRNA encoding Cdh1 and no reduction in the Cdh1 protein levels. The ratio of cell surface-to-cytoplasmic Cdh1 distribution was not different in the prechordal plate cells between control and ethanol treated embryos. However, there were cytoplasmic Cdh1 aggregates in the ethanol treated embryo prechordal plate cells, the significance of with remains unclear. The evidence indicated that there was no significant difference in Cdh1 levels or distribution [6].

Gene expression analysis comparing 8 hpf embryos treated with ethanol as compared with control embryos showed numerous ethanol dysregulated genes. One highly dysregulated gene was that encoding protocadherin-18a (Pcdh-18a), being reduced nearly 2-fold. We validated this gene expression change using quantitative PCR [6]. Protocadherins were shown to partner with classical cadherins to promote normal cell adhesion [39]. Perhaps reduced Pcdh-18a is responsible for aspects of the epiboly and gastrulation defects that resemble Cdh1 loss-of-function. This was tested by injecting synthetic mRNA encoding Pcdh-18a into embryos, which restored more normal epiboly and convergent extension phenotypes in ethanol and mRNA injected embryos that more closely resemble control embryos [6]. Together, our data showed that adhesion regulation was disrupted in ethanol exposed early embryos, producing gastrulation defects.

### 4.3. Sonic Hedgehog

Several studies have implicated sonic hedgehog (Shh) signaling defects in ethanol-induced birth defects. The hedgehog (Hh) family of proteins are embryonic morphogens that mediate signal transduction pathways, regulating cell specification, differentiation, and help maintain stem cells [17,40]. They form a spatial gradient in the tissue environment, inducing differential gene expressions in a concentration dependent manner [17]. Shh, one of the three members of the Hh family in vertebrates, plays a critical role in embryonic cell proliferation, differentiation, and morphological patterning [40].

Shh processing regulates ligand secretion and, thus, signaling. Posttranslational lipid and cholesterol modification of Shh occurs in the Golgi. Modified Shh forms a protein complex with caveolin (Cav1), allowing for intracellular vesicular transport to lipid rafts in the plasma membrane, where it is then secreted [40,41]. In vertebrates, extracellular modified Shh binds to patched (Ptc), releasing smoothened (Smo) from the receptor complex. Smo signaling decouples suppressor of fused (SuFu), a negative regulator, from glioma-associated oncogene (Gli), allowing Gli to enter the nucleus and activate transcription [17,40].

Ethanol exposure during development can induce holoprosencephaly (HPE) [42,43]. HPE is characterized by defective rostroventral midline patterning of the forebrain with an array of other abnormalities, including failure of the forebrain to form hemispheres and cyclopia [44,45]. Disruption to various points in the Shh pathway, with or without exposure to ethanol, during development, can also lead to HPE [10,40,44,46].

Ethanol treated embryos have a similar phenotype to embryos deficient in *shh*, leading to the hypothesis that *shh* function is affected by ethanol exposure [10,40,46,47]. When exposed to alcohol, a defective posttranslational cholesterol modification on Shh may lead to reduced Shh signaling [40]. Studies have also shown that phenotypes produced by embryonic ethanol exposure such as cyclopia and other midfacial defects can arise from cell death of neural crest cells. Furthermore, *shh* developmental signaling was indirectly displaced by a synergistic interaction between ethanol and cyclopamine, a *shh* pathway inhibitor [43]. A rescue experiment using *shh* mRNA injection into ethanol treated zebrafish embryos reduced ethanol induced phenotypes, indicating that *shh* signaling is disrupted in FAS [47].

### 4.4. Cdon

In some patients with HPE, a loss of CDON (gene name for cell adhesion associated, oncogene regulated) function, a cell surface protein that facilitates the Shh pathway, was identified [45,46]. *Cdon* is a multi-functional co-receptor for Hh receptor and other receptor proteins [45,48]. Loss of *Cdon* in mice results in a mild HPE phenotype, but when coupled with ethanol exposure, more severe HPE phenotypes develop [49]. Similarly, a zebrafish study using *cdon* targeted morpholinos, *cdon* expression knockout produced mild craniofacial hypoplasia and did not produce and increase in cell death [50]. It was also shown that manipulation of the Shh pathway in zebrafish affected *cdon* expression during neural crest cell migration and epithelial mesenchymal transition, indicating that *cdon* responds to Shh signals [50]. Another study looked at the effect of Cdon in zebrafish eye development and found that Cdon functions as a negative regulator of Hh signaling in proximal-distal eye patterning [51].

Cdon also physically and genetically interacts with the Nodal pathway in mice, though the mechanism is still unclear [45]; Nodal signals prechordal plate (PCP) development from the anterior primitive streak, and the PCP produces Shh, which initiates forebrain patterning and rostroventral midline development [45]. Therefore, a defect in Nodal signaling during primitive streak formation, due to mutation or ethanol exposure, can exacerbate Hh signaling defects and HPE [45]. This study in mice suggests that Cdon plays an early role in development, prior its role as an Shh co-receptor.

Cdon may be redundant with other co-receptors like LRP2 [45]. Cdon and Lrp2 were shown to have similar functions in Nodal signaling. Mouse double mutants in Cdon and Lrp2 showed similar phenotypes as Nodal pathway mutations [45]. Additional studies are needed to understand ethanol effects on Nodal pathway and its downstream effects on Shh signaling during early development. Zebrafish may be useful to dissect the Nodal and Shh signaling pathway interactions and their interactions with ethanol.

## 5. Nodal and Bmp Gradients

Nodal and bone morphogenetic protein (Bmp) are transforming growth factor-*ß* (TGF-*ß)* superfamily members that regulate DV axis establishment [52,53,54]. Nodal and Bmp together, forming gradients in the DV axis of the zebrafish embryo consistent with the source/sink signal dispersal model hypothesized by Francis Crick in 1970 [55]. This model states that a signal is constantly produced at a localized source and diffuses through tissue where it is then destroyed or inhibited by a localized sink at specific distance away, forming a gradient signal that regulates morphogenesis [54,55]. Nodal and Bmp fit this model perfectly. Nodal signaling is concentrated on the dorsal end and Bmp signaling on the ventral end, both diffusing into the center of the embryo and inhibitors suppress further activation in distant areas [54,55].

Nodal and Bmp are first expressed from maternal transcripts in the YSL of a developing zebrafish embryo [13]. Nodal functions through two Nodal related genes, *nrd1* and *nrd2*, and its inhibitor, Antivin, to specify mesoderm and endoderm (mesendoderm) progenitors [13,52,56]. The ventral mesendoderm is formed receiving Bmp signals on the ventral-most side of the embryo. Chordin inhibits Bmp and is secreted by the dorsal organizer on the dorsal-most side of the embryo, which binds directly to Bmp to block its receptor interaction and signaling [52,54,57]. In zebrafish, the maternal Wnt/*ß*-catenin pathway activates the Nodal/Bmp cascade as well as Oct4, Drap1, and FoxH1 targets of Nodal signaling [56,58,59].

**Figure 1:**
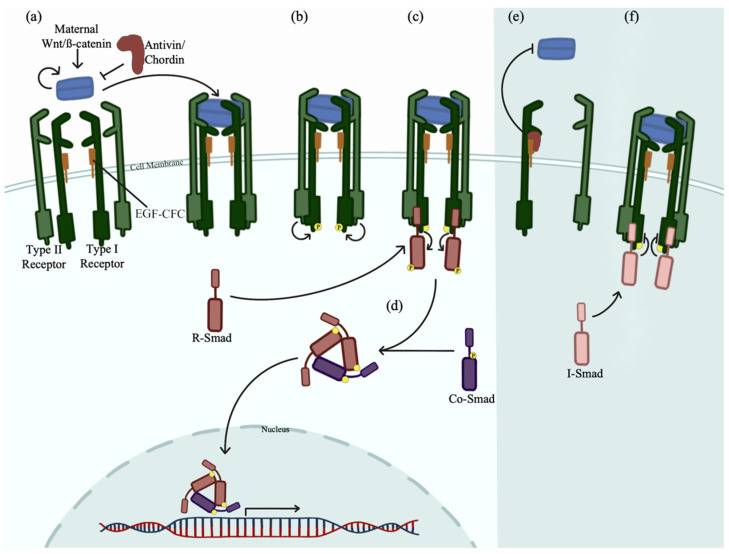
TGF-ß Signaling. TGF-ß ligands Nodal and Bmp activate Smad signaling. (**a**) Maternal Wnt/ß-catenin signals Nodal or Bmp ligands. These ligands bind and assemble the type I and type II heteromeric receptor complex. EGF-CFC co-receptors are bound to type I receptors. (**b**) Ligand binding transduces the signals intracellularly. Type II receptors phosphorylate type I receptors signaling Smad proteins. (**c**) R-Smad proteins are phosphorylated by type I receptors. Nodal specific R-Smads are Smad2 and 3. Bmp specific R-Smads are Smad1, 5, and 9. (**d**) Two R-Smads complex with one phosphorylated co-Smad4 and translocate into the nuclease and activate gene transcription. (**e**) Antivin or Chordin inhibits Nodal or Bmp, respectively, by binding to the EGF-CFC co-receptor on the type I receptor blocking ligand binding to the receptor. (**f**) After receptor phosphorylation, I-Smad6 or 7 can bind to the receptor complex blocking R-Smad binding and signaling.

TGF-ß ligands, Nodal or Bmp, in their respective cellular locations, bind to and assemble the type I and type II activin transmembrane serine/threonine kinase receptor complex (Figure 1a) [60,61]. The heteromeric receptor complex, containing two type I and two type II receptors transduces the signal intracellularly by binding and phosphorylating receptor-regulated cytoplasmic Smad proteins (R-Smads) (Figure 1b,c). Common mediator Smad4 (Co-Smad) assembles with R-Smads in the cytoplasm forming heterotrimeric complexes which are then translocated into the nucleus to activate gene expression [54,62,63,64,65]. Nodal signaling leads to Smad2/3 phosphorylation, and Bmp signaling activates gene transcription through Smad 1/5/9 phosphorylation (Figure 1d) [54,62].

The TGF-ß pathway can be inhibited by Antivin or Chordin binding to EGF-CFC (epidermal growth factor- Crypto, FRL1, Cryptic) membrane linked coreceptor glycoproteins, blocking ligand signaling (Figure 1e) [54,57,66,67]. Inhibitory Smads (I-Smads), Smad6 and Smad7, can also inhibit this pathway by preventing intracellular Smad signaling by associating with the type I receptor (Figure 1f) [53,68,69,70].

A 2010 study using a 3% ethanol treatment for 3 h on mid-to late-blastula stage embryos showed a split axis phenotype starting at 24 hpf [71]. This phenotype resulted from cell movement disruption during the blastula and gastrulation stages [71]. This suggests that marginal tissue from the blastopore organize axis formation, but ethanol exposure delays epiboly progression, allowing a premature marginal axis to form and producing a split axis phenotype [71].

## 6. Conclusions

Ethanol has detrimental effects on a developing embryo, and the zebrafish is a useful model for a developing human exposed to ethanol in utero (FASD) [8,25]. The range of defects depend on the concentration of ethanol and the timing of exposure, which produce developmental delays, brain defects, heart defects, craniofacial abnormalities, and potential lethality [1,2,9]. Ethanol affects transcriptional activity, but there may be independent effects within cells on proteins, protein complexes, lipid membrane structures and other effects. For example, our laboratory showed effects on the microtubule cytoskeleton in the yolk cell that occurred within 1 h of ethanol exposure at or prior to zygotic genome activation [6].

Our lab has previously studied the effects of embryonic ethanol exposure from the pre-gastrulation and mid-gastrulation stages, using Affymetrix GeneChip microarray gene expression analysis [6,25]. Ethanol exposure during embryogenesis alters the expression of important developmental genes. Sox2, Elf3, and their transcriptional targets produced potential ethanol dysregulated gene regulatory network changes [25]. Additional study is needed to understand the consequences of this gene regulatory network dysregulation.

A study by Hong et al., examined ethanol exposure effects on Nodal signaling using a Cdon mutation in a mouse model, showing ethanol effects on the interactions and trafficking of signaling proteins instead of directly disrupting early gene expression [49]. Many important early development activities are expressed from maternal transcripts in the early embryo [13]. If ethanol is altering these signaling activities, it could help explain the FASD phenotype that includes effects on the neural and body axes.

Pleiotropic effects of embryonic ethanol exposure make it difficult to sort out mechanisms. Furthermore, there are relatively few studies of ethanol exposure on the early embryo. The zebrafish is a particularly useful model for studying early development, like the experiments characterizing ethanol effects on cell adhesion and gene expression during zebrafish epiboly and gastrulation.

## Data Availability

Not applicable.
